# Determinants of Health Service Responsiveness in Community-Based Vector Surveillance for Chagas Disease in Guatemala, El Salvador, and Honduras

**DOI:** 10.1371/journal.pntd.0003974

**Published:** 2015-08-07

**Authors:** Ken Hashimoto, Concepción Zúniga, Eduardo Romero, Zoraida Morales, James H. Maguire

**Affiliations:** 1 Chagas Disease Control Project, Japan International Cooperation Agency, Tegucigalpa, Honduras; 2 National Chagas Disease Control Program, Directorate of Health Promotion, Ministry of Health, Tegucigalpa, Honduras; 3 National Vector-Borne Diseases Control Unit, Directorate of Environmental Health, Ministry of Health, San Salvador, El Salvador; 4 National Vector-Borne Disease Control Program, Department of Regulation of Healthcare Programs, Ministry of Health, Guatemala City, Guatemala; 5 Brigham and Women´s Hospital, Harvard Medical School, Boston, Massachusetts, United States of America; Universidad Autónoma de Yucatán, MEXICO

## Abstract

**Background:**

Central American countries face a major challenge in the control of *Triatoma dimidiata*, a widespread vector of Chagas disease that cannot be eliminated. The key to maintaining the risk of transmission of *Trypanosoma cruzi* at lowest levels is to sustain surveillance throughout endemic areas. Guatemala, El Salvador, and Honduras integrated community-based vector surveillance into local health systems. Community participation was effective in detection of the vector, but some health services had difficulty sustaining their response to reports of vectors from the population. To date, no research has investigated how best to maintain and reinforce health service responsiveness, especially in resource-limited settings.

**Methodology/Principal Findings:**

We reviewed surveillance and response records of 12 health centers in Guatemala, El Salvador, and Honduras from 2008 to 2012 and analyzed the data in relation to the volume of reports of vector infestation, local geography, demography, human resources, managerial approach, and results of interviews with health workers. Health service responsiveness was defined as the percentage of households that reported vector infestation for which the local health service provided indoor residual spraying of insecticide or educational advice. Eight potential determinants of responsiveness were evaluated by linear and mixed-effects multi-linear regression. Health service responsiveness (overall 77.4%) was significantly associated with quarterly monitoring by departmental health offices. Other potential determinants of responsiveness were not found to be significant, partly because of short- and long-term strategies, such as temporary adjustments in manpower and redistribution of tasks among local participants in the effort.

**Conclusions/Significance:**

Consistent monitoring within the local health system contributes to sustainability of health service responsiveness in community-based vector surveillance of Chagas disease. Even with limited resources, countries can improve health service responsiveness with thoughtful strategies and management practices in the local health systems.

## Author Summary

Elimination of domiciliated vectors led to a decreased prevalence of Chagas disease in parts of Latin America. In Central America, where the domiciliated vector *Rhodnius prolixus* has been almost eliminated, *Triatoma dimidiata*, which cannot be eliminated, continues to threaten the population in vast areas. To maintain the risk of transmission of *Trypanosoma cruzi* at lowest levels despite limited resources, Guatemala, El Salvador, and Honduras integrated community-based vector surveillance into local health systems. One challenge to sustaining surveillance is to ensure continuous responsiveness to reports of household infestation from the community. Our research in 12 study areas in the three countries over a five-year period investigated eight potential determinants of health service responsiveness, including volume of vector notifications, local geography, demography, manpower, and managerial approach. We found that consistent (quarterly) monitoring by departmental personnel within the local health services was associated with high response rates. Results of interviews added additional insight.

## Introduction

The prevalence of Chagas disease in Central America decreased from 1.7 million in the 1990s to 0.4 million in 2010 as a result of successful vector control [1, 2]. Of the two main vectors, *Rhodnius prolixus* is almost eliminated, but *Triatoma dimidiata* remains widespread in the region despite greatly reduced rates of household infestation [3–8]. To prevent transmission of Chagas disease resulting from re-infestation of houses by *T*. *dimidiata* in areas with limited resources, Guatemala, El Salvador, and Honduras implemented community-based surveillance, in which community members report the presence of bugs in houses to trigger a response by local health services of the Ministry of Health [5, 9, 10].

Community-based surveillance has been shown to be effective and cost-effective, but can be challenging to sustain [11–14]. Household infestation with vectors can be detected readily by inhabitants, and in turn, health services are expected to respond to every vector report by visiting houses to spray insecticide and provide educational advice [9, 11]. However, little is known about the extent to which vector reports from the community are met with appropriate responses and the factors that determine responsiveness of health services to vector reports.

Research on responsiveness of health services may provide insights that help sustain and strengthen vector surveillance throughout the region. We retrospectively analyzed health services’ response rates and underlying determinants in community-based vector surveillance of Chagas disease in Guatemala, El Salvador, and Honduras.

## Methods

### Study areas

**Fig 1:**
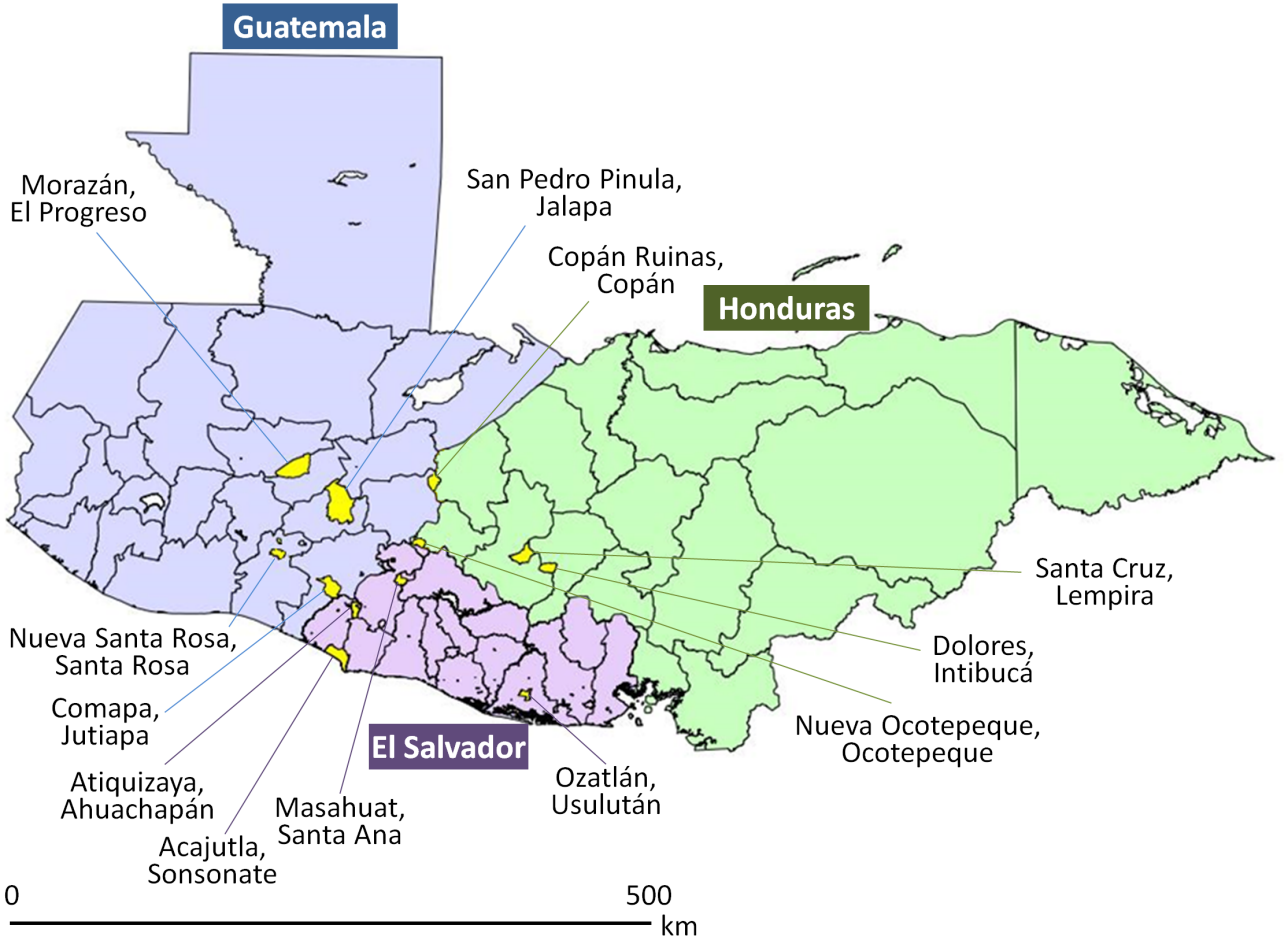
Location of the 12 study areas in Guatemala, El Salvador, and Honduras.

We selected 12 areas with community-based vector surveillance in Guatemala, El Salvador, and Honduras–four from each country (Fig 1). Each area was a conglomerate of villages and defined as being under the jurisdiction of a particular health center. For inclusion of an area in the study, the Ministry of Health had to have completed the attack phase in all villages by conducting multiple cycles of extensive insecticide spraying of at-risk houses to reduce household vector infestation, implemented community-based vector surveillance, and recorded data from 2008 to 2012. To compare management styles in unevenly decentralized health systems, we included one area per Department. The selected study areas were rural and in the most endemic districts of the Departments.

**Fig 2:**
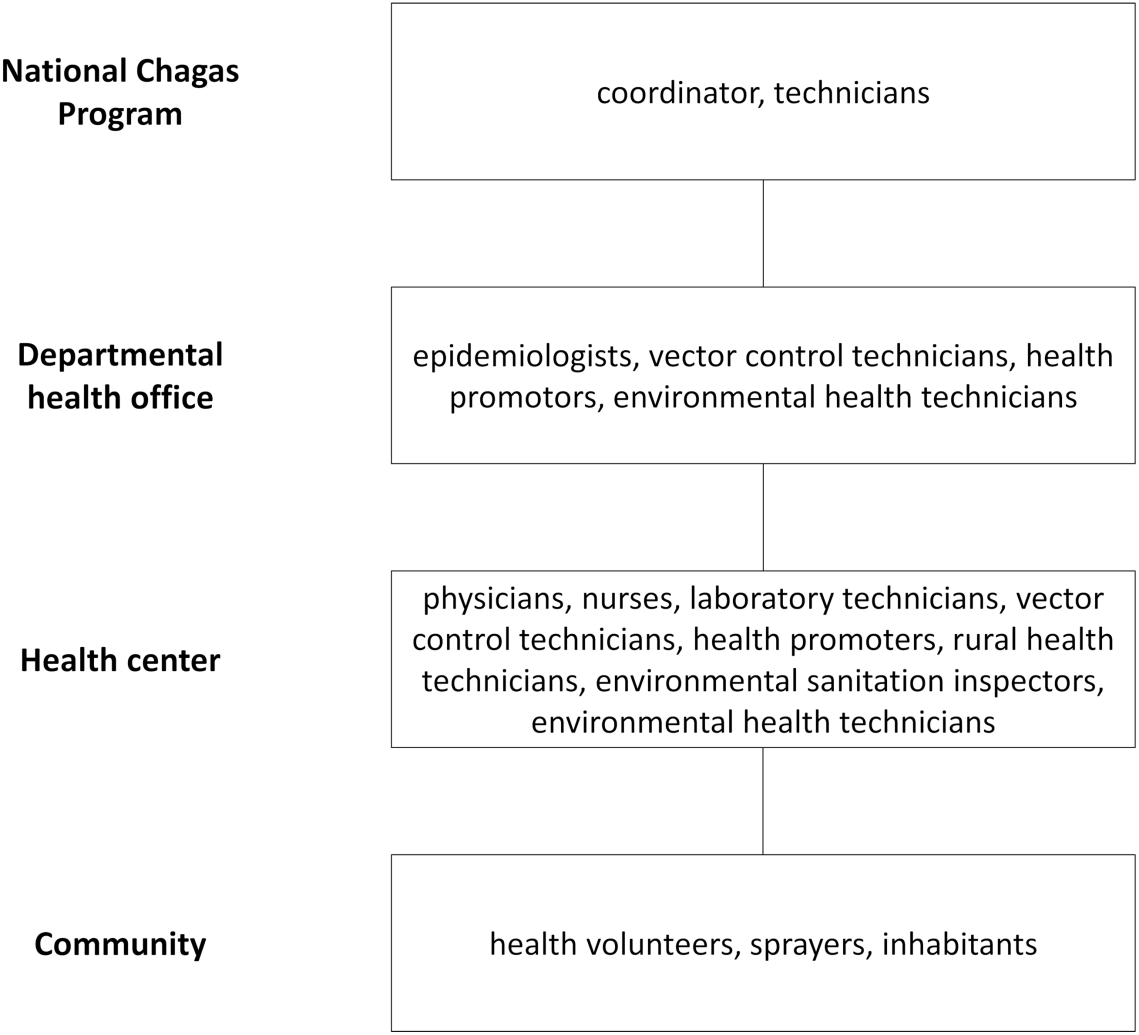
Actors involved in Chagas disease vector surveillance in Guatemala, El Salvador, and Honduras.

The study areas varied in population size (1,160 to 33,579 persons), geographic area (6 to 150km^2^), entomological situation, and human resources (Table 1 and Fig 2). The main target vector for surveillance in the study areas was *Triatoma dimidiata*, although in six areas (two in Guatemala and four in Honduras) surveillance also focused on *Rhodnius prolixus* because of previous history of infestation.

**Table 1:** Demography, geography, and human resources in the 12 study areas in Guatemala, El Salvador, and Honduras.

	Guatemala				El Salvador				Honduras			
**Department**	Santa Rosa	El Progreso	Jutiapa	Jalapa	Santa Ana	Ahuachapán	Usulután	Sonsonate	Ocotepeque	Copán	Intibucá	Lempira
**Municipality**	Nueva Santa Rosa	Morazán	Comapa	San Pedro Pinula	Masahuat	Atiquizaya	Ozatlán	Acajutla	Nueva Ocotepeque	Copán Ruinas	Dolores	Santa Cruz
**Health center**	Ojo de Agua	Morazán	Comapa	San Pedro Pinula	Masahuat	Atiquizaya	Ozatlán	Metalio	San José de la Reunión	Rincón del Buey	Dolores	Santa Cruz
**Population**	2,384	12,228	28,991	43,092	5,499	33,579	12,733	13,326	1,160	5,053	5,600	6,857
**Number of households**	749	3,018	4,934	9,132	1,480	8,699	3,999	4,338	140	865	1,100	1,245
**Number of villages**												
total	1	80	62	21	23	106	41	36	6	13	19	33
with history of *T*. *dimidiata*	1	35	62	21	20	75	17	36	6	11	19	12
with history of *R*. *prolixus*	0	0	4	17	0	0	0	0	6	3	8	5
with vehicle access	1	75	58	21	23	106	41	36	6	10	19	27
**Geography**												
Area of jurisdiction (km^2^)	6	31	132	85	71	40	50	66	14	34	82	150
Distance from health center to departmental capital (km)	22	31	40	20	42	12	12	20	17	123	50	65
**Number of health personnel**												
Physicians	1	1	5	7	3	7	3	3	0	1	1	1
Nurses	0	12	3	29	6	19	4	6	1	1	2	3
Laboratory technicians	0	1	1	1	1	3	1	0	0	0	0	0
Vector control technicians	1^a^	2^b^	5	4^b^	0	1	2^b^	2^b^	0	0	0	0
Other operational technicians	1^a^	1	6	6	10	30	11	19	1^a^	1^b^	1	1^b^
Vector control & operational technicians (total)^c^	1	2	11	8	10	31	12	21	0.5	0.5	1	0.5
Community health volunteers	5	4	25	28	20	186	64	36	30	26	48	43
Community sprayers	0	0	17	0	10	12	2	22	6	11	10	20

^a^ Technicians assigned to another local health center covered the health centers in the study areas by regular visits.

^b^ Technicians assigned to the departmental office covered the health centers in the study areas by regular visits.

^c^ Technicians not directly assigned to the health centers (a and b) were counted as 0.5 persons.

All 12 health centers had physicians, nurses, and operational technicians except San José de la Reunión in Honduras, which had no physicians, and Ojo de Agua in Guatemala, which had no nurses (Table 1). Operational technicians had different qualifications or responsibilities. Vector control was carried out by vector control specialists and occasionally assisted by unspecialized rural health technicians in Guatemala; and was jointly conducted by vector control specialists, health promoters, and environmental sanitation inspectors in El Salvador. In Honduras, environmental health technicians were responsible for food security, environmental sanitation, and zoonoses as well as vector control. Some technicians belonged to neighboring health centers or a departmental office, and covered the health centers through regular visits. Community health volunteers were present in all 12 health centers and insecticide sprayers were present in nine.

### Vector surveillance in study areas

#### Implementation

The attack phase of Chagas disease control was carried out in Guatemala from 2000 to 2005 and in El Salvador and Honduras from 2004 to 2007. Although the same spraying techniques, equipment (Hudson X-pert), and insecticides (pyrethroids, mostly deltamethrin 5% wp) were used, the sprayers were vector control technicians in Guatemala, vector control technicians and health promotors in El Salvador, and paid trained community personnel in Honduras. Within two to three years of completing the attack phase, the three countries implemented community-based vector surveillance. Because communities had participated in searching and sending vector bugs to the local health centers during the attack phase, surveillance activities were familiar to most of the population in the study areas. The attack and surveillance phases were implemented with technical assistance of the Japan International Cooperation Agency (JICA) through bilateral projects (Guatemala during 2000–2005 and 2009–2012; El Salvador and Honduras during 2004–2011).

The National Chagas Program in each country designed a surveillance model and trained the personnel of the departmental health offices, who in turn trained the health center staff. The departmental or health center personnel oriented and provided community health volunteers with health promotion materials (e.g. posters, brochures, T-shirts), and trained community sprayers.

#### Structure and management

**Fig 3:**
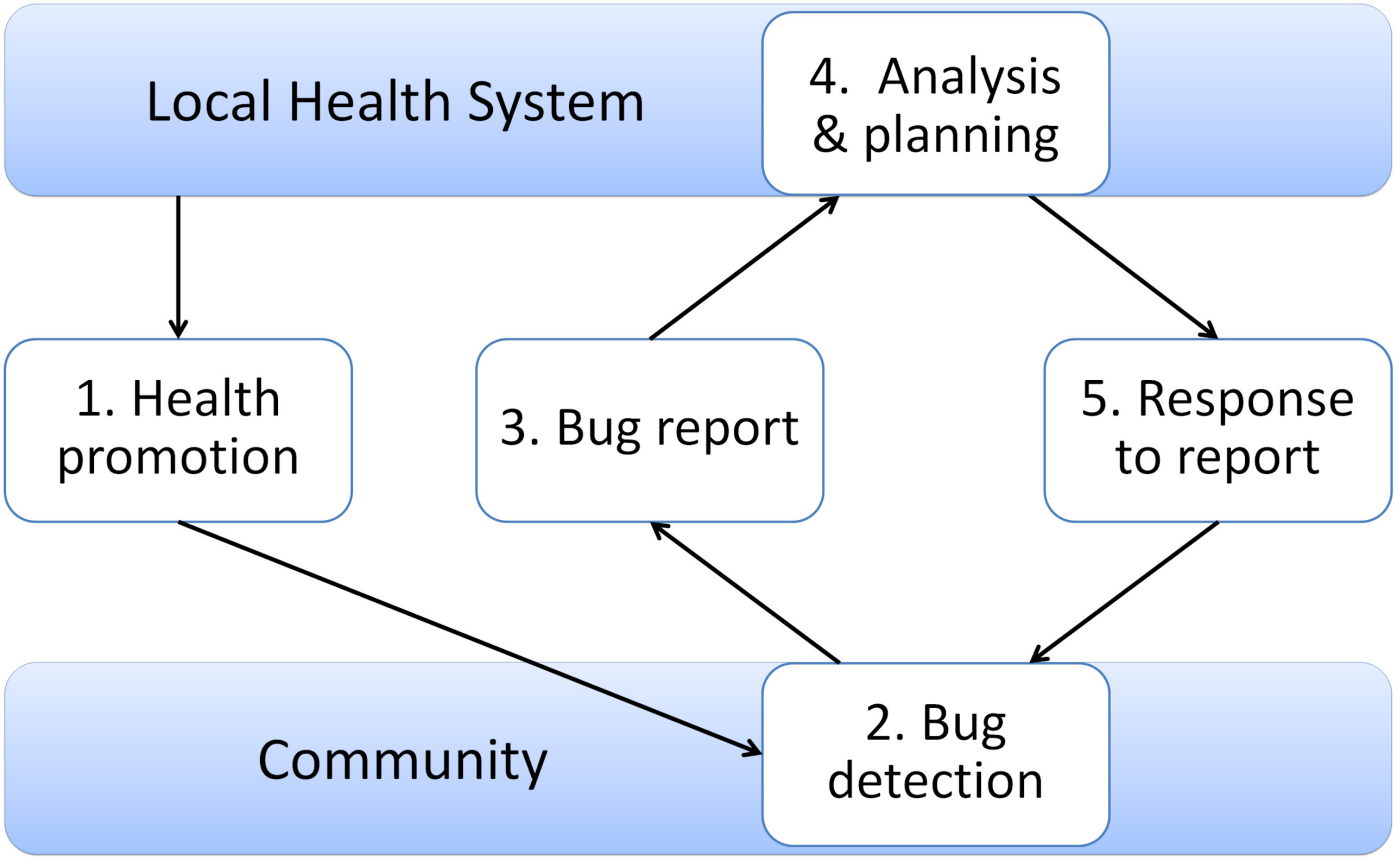
Five essential functions of Chagas disease community-based surveillance [9].

Community-based vector surveillance for Chagas disease consisted of five essential functions: 1) health promotion—instruction of the community on how to search for bugs; 2) detection of bugs in houses by inhabitants; 3) reporting of bugs to health centers; 4) analysis of reports of bugs, and decision making and planning for response; and 5) response to the report (Fig 3) [9]. Health center staff and community health volunteers promoted bug searches through different networks including nuclear and extended families, neighborhoods, schools, and churches. Community health volunteers were given labels to attach to bug containers (usually plastic bags or plastic bottles) that displayed the address of infested households, name of head of household, and detection date of each infested household. Inhabitants searched for vectors in houses and, if detected, placed them in labelled bug containers and then in a deposit box on the premises of health centers (often in the waiting room) or occasionally in schools or community health volunteers’ houses for later transfer to health centers or collection by local operational technicians. Trained health personnel at the health center or departmental health office registered vector reports (except those identified as non-vector insect species), analyzed data, and made decisions for response.

The three countries responded to bug notifications either by insecticide spraying or educational advice, according to different criteria. In Guatemala and Honduras, households were sprayed if infested with nymph(s) or high densities of *T*. *dimidiata* (generally more than three specimens per household). In El Salvador all infested households were eligible for spraying, although national guidelines allowed educational advice instead of spraying. Insecticide spraying was carried out principally by vector control technicians in Guatemala, by vector control technicians, health promoters, environmental sanitation inspectors, or trained community personnel in El Salvador, and by trained community personnel in Honduras. The responsible individuals applied pyrethroid insecticide (deltamethrin 5% wp) using a manual pump sprayer (Hudson X-pert) and spraying techniques as employed in the attack phase. Guidelines mandated a response to bug reports within seven days in El Salvador and within one month in Guatemala and Honduras. However, the time to response often exceeded one month because of lack of immediately available sprayers, transportation, or insecticide, and when there were dengue outbreaks.

Departmental technicians monitored community-based surveillance by periodic visits (quarterly on average) to the health centers to review the registry, check spraying equipment, participate in monthly meetings with community health volunteers, and exchange information with the local health personnel. During these visits, they would also supervise and review activities of insecticide spraying and its effects.

### Study design

We defined a health service’s response rate as the percentage of the number of households sprayed or visited for advice by the local health services divided by the number of households infested with Chagas disease vectors as reported by the community. The annual response rate was calculated for each study area between 2008 and 2012, so that a total of 60 response rates (12 areas x 5 years) potentially were available for analysis. If the response occurred during the year following notification, it was considered as an action of the year of notification. A household with consecutive notifications of vector infestation was counted as a single infested household until the health service responded, regardless of interval length between notification and response.

Taking in account factors that might influence demand, supply, and work process in community-based vector surveillance, we selected for analysis the following eight variables as potential determinants of health service responsiveness: number of infested households as reported by the community; distance from health centers to departmental capitals; number of operational technicians per 1,000 households; numbers of community volunteers and sprayers per 1,000 households; interval between receipt of vector reports from the community and response by health services, i.e. <3 months, 3–12 months or >12 months; degree of decentralization of response to vector reports, i.e. by health center or departmental office; presence of consistent monitoring by departmental technicians; and presence of technical assistance by JICA.

We collected data on surveillance activities, local demography, geography, and human resources during visits to the departmental health offices and health centers during 2013. We interviewed personnel responsible for Chagas disease vector surveillance in each facility to identify any perceived factors or circumstances that might have influenced responsiveness during the five year period.

### Data analysis

#### Statistical analysis

We conducted multivariable linear regression to assess the relationship between the response rate and potential determinants. We further analyzed the data using mixed-effects multi-linear regression, clustering the yearly response rates by health center, to account for differences between and within the health centers. All statistical analyses were conducted using STATA version 12 software.

#### Qualitative analysis

The results of interviews at the 12 health centers and corresponding departmental health offices were analyzed to explain differences in the response rates. We also analyzed the roles of participants (stakeholder analysis) within the five essential functions of community-based surveillance (Fig 3) [9] to identify responsible personnel, organizational patterns, and managerial focal points in each study area.

## Results

### Descriptive characteristics of study areas

Communities reported a total of 2,630 households with *T*. *dimidiata* infestation in the 12 study areas between 2008 and 2012. Of these, the Ministry of Health responded to 2,041 households (response rate 77.6%, Table 2 and S1 Table). Of the 2,041 responses, 68.4% were by insecticide spraying and the reminder by providing education and advice.

**Table 2:** Results of community-based vector surveillance by country in the 12 study areas in Guatemala, El Salvador, and Honduras from 2008 to 2012.

Surveillance indicators		2008	2009	2010	2011	2012	Total
**Number of households reported with vector by the community (N = 57)**	Guatemala	159	323	138	82	374	1,076
	El Salvador	106	121	173	229	199	828
	Honduras	102	459	67	61	37	726
	TOTAL	367	903	378	372	610	2,630
**Mean health services' response rate (%) (N = 56)**	Guatemala	79.2	78.9	55.1	74.4	75.9	74.5
	El Salvador	92.5	87.6	80.3	97.4	86.4	89.1
	Honduras	26.5	77.1	68.7	83.6	62.2	69.0
	MEAN	68.4	79.2	69.0	90.1	78.5	77.6
**Percentage of responses with insecticide spraying** * **(%) (N = 56)**	Guatemala	18.3	22.0	76.3	52.5	35.2	33.5
	El Salvador	87.8	99.1	96.4	97.3	95.8	95.8
	Honduras	100	96.3	47.8	52.9	17.4	84.0
	MEAN	54.2	70.2	82.0	82.4	56.2	68.4

See S1 Table for data by study area.

*(number of households sprayed / number of households responded) x 100

N = number of data sets; N was less than the maximum possible (60 = 12 study areas x 5 years) because of missing reports of bug notification in Ojo de Agua in Guatemala from 2008 to 2010 and in Rincón del Buey in Honduras in 2008.

Values of the eight variables that potentially influenced health service’s response rates differed among the health centers, but remained relatively constant within health centers over the 5-year period, with the exception of number of infested households reported, consistent monitoring by departmental technical officials, and technical assistance by the JICA project (Table 3). Numbers of health workers fluctuated according to trainees’ temporary assignments, and the population size of areas grew over time, but we treated these data as constant over the five year period.

**Table 3:** Potential determinants of health services’ response rates for community-based vector surveillance in the 12 study areas in Guatemala, El Salvador, and Honduras from 2008 to 2012.

**Potential determinants of health service responsiveness**	**N**	**Mean**	**Std. Dev**.	**Minimum**	**Maximum**
**Number of households reported with vector bugs**	57	46.1	68.7	0	356
**Distance from health centers to departmental capitals (km)** ^a^	60	37.8	14.0	12	123
**Number of operational technicians per 1,000 households** ^a^	60	2.2	4.2	0.2	6.8
**Number of community volunteers and sprayers per 1,000 households** ^a^	60	40.5	14.2	0.6	257
	**N**	**No**	**Yes**		
**Presence of technical assistance by JICA project**	60	37	23		
**Consistent monitoring by departmental technical officials**	60	9	51		
	**N**	**Health Center**	**Departmental office**		
**Degree of decentralization of response to vector reports**	57	37	20		
	**N**	**< 3months**	**3-12months**	**>12months**	
**Interval between receipt of vector report from the community and response by health services**	57	29	16	12	

^a^ Remained constant throughout the five year period of 2008–2012.

### Potential determinants of responsiveness

Of the eight variables analyzed, two were found by linear regression and mixed-effects multi-linear regression to be significantly associated with health service responsiveness: consistent monitoring by departmental technicians and technical assistance by JICA (Table 4). In both regression analyses, consistent monitoring from the departmental level was correlated positively with health service responsiveness to a moderate degree (*r* = 0.48–0.55) whereas the correlation of assistance from JICA was weak and negative (*r* = -0.13).

**Table 4:** Results of linear regression and mixed-effects multi-linear regression on potential determinants of health service responsiveness in community-based vector surveillance in Guatemala, El Salvador, and Honduras (N = 56).

	Linear Regression						Mixed Effects Multi-linear Regression Clustered by Health Center					
Potential determinants of institutional response coverage	Coefficient	Std. Error	t	*p*	95% Confident Interval		Coefficient	Std. Error	t	*p*	95% Confident Interval	
**Number of households reported with vector bugs**	-0.0008	0.0004	-1.72	0.092	-0.0017	0.0001	0.0000	0.0005	0.04	0.972	-0.0009	0.0010
**Distance from the health center to the departmental capital (km)**	-0.0022	0.0017	-1.30	0.201	-0.0056	0.0012	-0.0018	0.0020	-0.90	0.369	-0.0058	0.0021
**Number of operational technicians per 1,000 households**	0.0002	0.0169	0.01	0.990	-0.0338	0.0342	0.0008	0.0282	0.03	0.977	-0.0544	0.0560
**Number of community volunteers and sprayers per 1,000 households**	-0.0003	0.0005	-0.70	0.484	-0.0012	0.0006	-0.0002	0.0008	-0.23	0.822	-0.0017	0.0014
**Interval between receipt of vector report from the community and response by health services**	-0.0462	0.0531	-0.87	0.389	-0.1531	0.0606	-0.0322	0.0573	-0.56	0.574	-0.1445	0.0801
**Degree of decentralization of response to vector reports**	-0.1178	0.1015	-1.16	0.252	-0.3220	0.0865	-0.0958	0.1299	-0.74	0.461	-0.3503	0.1587
**Consistent monitoring by departmental technical officials**	**0.4801**	0.1349	3.56	**0.001**	0.2088	0.7513	**0.5514**	0.1141	4.83	**0.000**	0.3277	0.7750
**Presence of technical assistance by JICA project**	**-0.1282**	0.0579	-2.21	**0.032**	-0.2447	-0.0117	**-0.1334**	0.0579	-2.31	**0.021**	-0.2468	-0.0200

### Temporal variation of reports of infestation and health services’ response rates

Health centers in Dolores, Honduras and Comapa, Guatemala reported large numbers of infested households in 2009 and 2012, following campaigns in schools to promote bug searches as explained during interviews with health center staff (S1 Table).

Response rates followed four general patterns over the 5-year period: 1) nearly 100% response for most of the period, 2) nearly 100% for years 1 to 3 but then falling, 3) fluctuating moderately (between 50% and 100%), and 4) fluctuating substantially (between 0% and 100%) but with a tendency towards improvement.

When mixed-effects multi-linear regression was clustered by response pattern, similar associations between health services’ response rates and regular monitoring by departmental technicians (*r* = 0.71, *p*<0.01) and assistance from JICA (*r* = -0.15, *p*<0.01) were seen as in earlier models (S2 Table).

Interviews with health center personnel offered insight into the reasons underlying the different patterns (Table 5). Centers with higher response rates appeared to be more prepared to react to reports of infested houses; had better trained and more engaged workers; had superior management skills for coordinating and solving problems; and had greater support from higher institutional levels and local stakeholders such as community health volunteers and municipalities.

**Table 5:** Summary of interviews with health center staff to explain different patterns of response rates in community-based vector surveillance between 2008 and 2012.

1. Mostly 100%	2. Almost 100% to drop	3. Fluctuate 50%-100%	4. Fluctuate 0–100%
**San Pedro Pinula, Guatemala**	**Masahuat, El Salvador**	**Comapa, Guatemala**	**Morazán, Guatemala**
A team of vector control technicians responded immediately from the departmental capital travelling on motorbike.	The departmental vector control coordinator regularly monitored surveillance by the health center, but he retired in 2011.	A municipal vector control technician visited endemic villages on foot bimonthly to collect bugs and respond, but was frequently overwhelmed by the volume of bug reports	Response of the vector control team travelling from the departmental office was limited at times by availability of vehicle and fuel.
**Ozatlán, El Salvador**	**Rincón del Buey, Honduras**	**Atiquizaya, El Salvador**	**Metalio, El Salvador**
Departmental technicians trained and supervised sprayers recruited temporarily by the local municipality on a yearly basis.	A departmental technician, who monitored surveillance by the health center, left the position following a health system reform in 2010.	Operational technicians of the health center sprayed infested houses or trained community personnel to spray during monthly multipurpose visits, but were frequently overwhelmed by the volume of bug reports.	The departmental vector control team registered and responded to bug reports every few months, but after training a technician of the health center to consolidate bug report data, the response rate improved.
**San José de la Reunión Honduras**		**Santa Cruz, Honduras**	**Dolores, Honduras**
A trained nursing assistant registered bug reports and organized the response with community health volunteers and an operational technician, who visited monthly.		The head of health center, along with a departmental technician and trained community sprayers, organized responses only every one to two years because of lack of local operational staff.	Response rate dropped when an unmotivated technician was assigned for a year. For the remaining time, an operational technician investigated infested houses and organized community-wide spraying approximately every two years.

* Ojo de Agua in Guatemala was not included in the analysis due to lack of data on response rate from 2008–2010.

### Stakeholders and their roles in surveillance system

**Fig 4:**
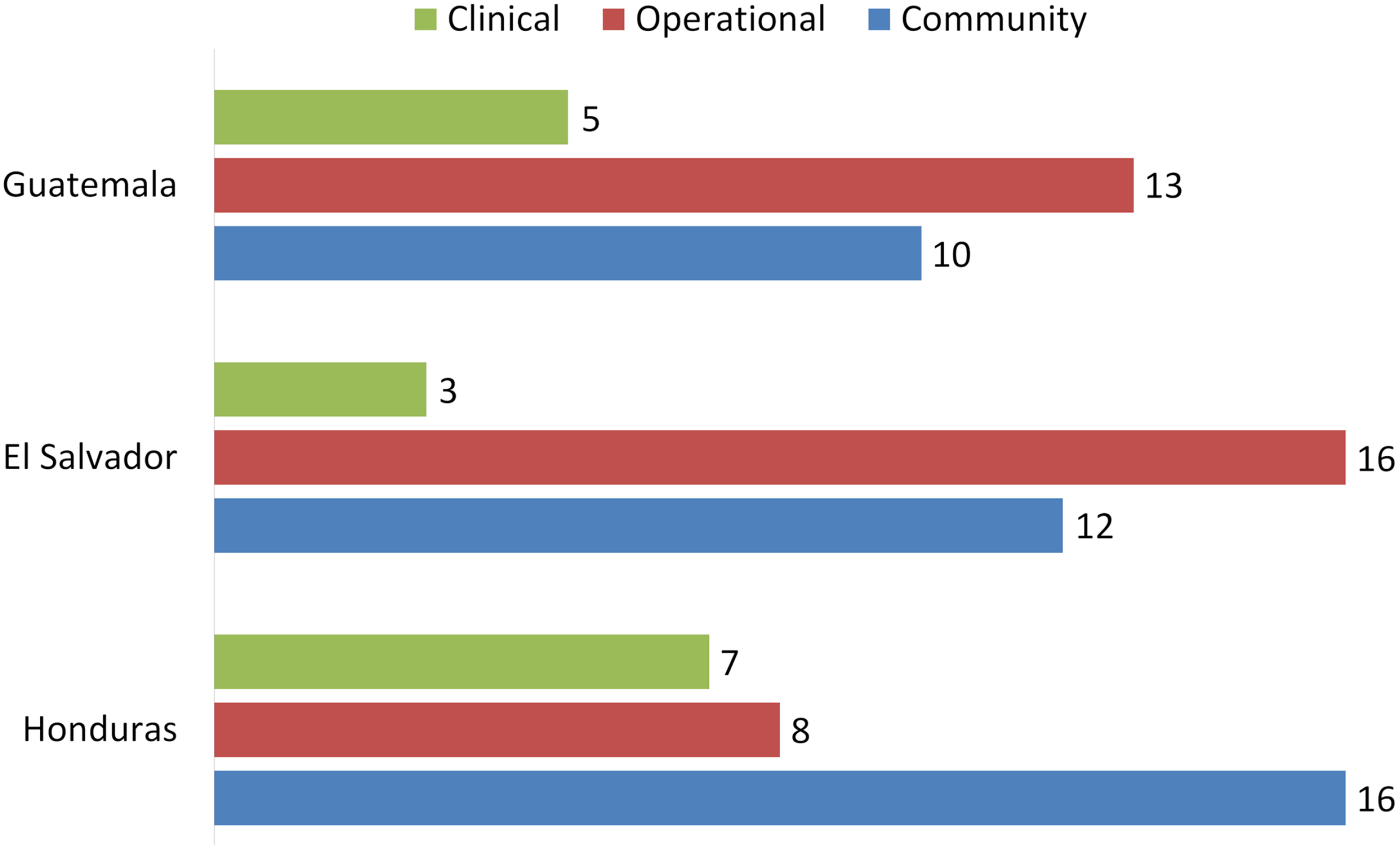
Number of clinical, operational, and community personnel in the community-based vector surveillance model with five essential functions in Table 6.

Interviews with the personnel of health centers and departmental health offices identified the persons responsible for different surveillance functions (Table 6). Bug detection was performed by the population in all study areas. Operational technicians or clinical staff of health centers were responsible for analysis, decision making and planning of response in 7 of the 12 study sites, while personnel at the Department level carried out this function in the other 5 areas (Table 6). Health promotion, bug reporting, and response to reports were conducted by distinct combinations of stakeholders in the different study areas. Overall Honduras recorded higher degrees of involvement by community personnel and clinical staff and lesser involvement by operational technicians than Guatemala and El Salvador (Fig 4).

**Table 6:** Participants and their roles in the community-based vector surveillance of Chagas disease in the 12 study areas in Guatemala, El Salvador, and Honduras.

		Guatemala				El Salvador				Honduras			
**Department**		Santa Rosa	El Progreso	Jutiapa	Jalapa	Santa Ana	Ahuachapán	Usulután	Sonsonate	Ocotepeque	Copán	Intibucá	Lempira
**Municipality**		Nueva Santa Rosa	Morazán	Comapa	San Pedro Pinula	Masahuat	Atiquizaya	Ozatlán	Acajutla	Nueva Ocotepeque	Copán Ruinas	Dolores	Santa Cruz
**Health center**		Ojo de Agua	Morazán	Comapa	San Pedro Pinula	Masahuat	Atiquizaya	Ozatlán	Metalio	San José de la Reunión	Rincón del Buey	Dolores	Santa Cruz
**1. Health promotion**	Clinical	NRS	NRS				PHS, NRS		PHS, NRS	NRS	PHS, NRS	PHS, NRS	PHS, NRS
	Operational	OPT	OPT	OPT	OPT	OPT	OPT	OPT	OPT		OPT	OPT	
	Community	CHV								CHV	CHV	CHV	CHV
**2. Bug detection**	Clinical												
	Operational												
	Community	POP	POP	POP	POP	POP	POP	POP	POP	POP	POP	POP	POP
**3. Bug report**	Clinical												
	Operational			OPT		OPT	OPT	OPT	OPT				
	Community	CHV, POP	CHV, POP	CHV, POP	CHV, POP	POP	POP	POP	POP	CHV, POP	CHV, POP	CHV, POP	CHV, POP
**4. Analysis, decision making and planning**	Clinical	NRS	LAB						NRS	NRS	PHY		PHY
	Operational	OPT	OPT(D)	OPT	OPT(D)	OPT	OPT	OPT, OPT(D)	OPT, OPT(D)		OPT(D)	OPT	OPT(D)
	Community												
**5. Response to report**	Clinical												
	Operational	OPT	OPT(D)	OPT	OPT(D)	OPT	OPT	OPT(D)	OPT, OPT(D)		OPT	OPT	OPT(D)
	Community			POP		POP	POP	POP	POP	CHV	CHV	CHV	CHV

PHS = Physician, NRS = Nurse, LBT = Laboratory technicians, OPT = Operational technician of health center, OPT(D) = Operational technician of departmental health office, CHV = Community Health Volunteer, POP = Population

## Discussion

We found that regular (quarterly) monitoring by departmental health offices was a significant determinant of health service responsiveness in community-based vector surveillance of Chagas disease in Guatemala, El Salvador, and Honduras. Perhaps surprisingly, response rates were significantly higher among health centers without presence of technical assistance by the donor (JICA). However, this finding can be explained by the presence of JICA at early stages of planning and implementation of the surveillance program at each study area, during which time response rates were low or fluctuated but subsequently improved. Three-year bilateral projects to establish community-based vector surveillance began in 2008 in El Salvador and Honduras and in 2009 in Guatemala.

Health service responsiveness was independent of the volume of bug reports from the community, distance between health centers and departmental offices, numbers of operational technicians in the local health service and community workers, intervals between vector report and institutional response, and degree of decentralization of response.

Interviews with health center staff demonstrated the effectiveness of regular monitoring on responsiveness and a decline in response rates following the departure of departmental supervisors in two health centers. This finding confirms previous research on primary health care services in low-resource settings, which showed that work performance was not motivated by written guidelines but by monitoring [15]. Because monitoring in this study provided an opportunity for departmental technicians and health center staff to review surveillance data, check equipment and supplies, participate in meetings with community health volunteers, and exchange information and experiences, continuation of quarterly visits should maintain or improve work performance over time. On the contrary, the consequences of inadequate monitoring can be serious in the long run, as reported in Gran Chaco in Argentina, where failure to supervise community personnel caused dysfunction of vector surveillance and reemergence of Chagas disease transmission [12].

Interviews also shed light into the lack of association between the other potential determinants and health service responsiveness. Although greater numbers of vector reports, for example following campaigns at school, increased the workload of local health services, response rates did not decline because manpower was augmented to meet the demand and tasks were reassigned among local stakeholders. Departmental technicians temporarily increased response capacity by mobilizing operational staff from other districts (as often occurs in reaction to dengue outbreaks) and by organizing extensive spraying operations with health center staff and community sprayers from different villages in the jurisdiction. Stakeholder analysis showed that surveillance tasks normally carried out by health specialists were simplified by the National Chagas Program and shifted to less specialized personnel through training, as we and others have reported previously [9, 16, 17]. In short, such combinations of short-term and long-term strategies reinforced responsiveness of health services.

Managerial responsibility for response at the departmental office rather than the health center did not appear to affect the response rate. Although the departmental response approach was more vertical and less integrated into primary health care services, interviews showed that both departmental health offices and health centers with high responsiveness were able to find solutions for difficult situations. For instance, departmental vector teams assigned a data collection technician to health centers, concentrated response efforts in time and space, and travelled by motorcycles to reduce transportation costs. A physician and a nurse at one health center posted a large map of the jurisdiction on a billboard in the waiting room and used thumbtacks to represent the number of households reporting vectors in each village and removed them following the appropriate response. Such strategies reinforced the management capacity of the local health services.

Longer intervals between receipt of vector reports and health service response did not lead to either higher response rates because of greater efficiency from economies of scale, or to lower response rates due to increased demands to deal with greater number of bug notifications. However, longer intervals are worrisome because of extended time of exposure of the population to the vector and thus greater risk of transmission of infection. Another potential negative impact is that the community may become reluctant to participate in bug notification if the interval is perceived as too long.

While portraying the reality of vector surveillance in Guatemala, El Salvador, and Honduras, this observational study has important limitations. The sensitivity of the analysis may have been affected by the limited number of infested households in certain areas and during specific years, and by lack of data at the individual household level, which would have detected repeatedly infested and responded households. Our resources were insufficient to measure outcomes such as household vector infestation rates and incidence and prevalence of Chagas disease. These data would enable analysis of the consequences of not achieving 100% response rate; the effect of spraying vs. educational advice; and the impact of variable quality of responses by specialized vs. lay workers. We were unable to conduct cost analyses that would allow us to compare the effectiveness of the different styles and approaches to integrated surveillance, which varied substantially among the 12 study areas [12]. Further research is needed to address these limitations as well as long-term effects of monitoring on community-based surveillance where stakeholders may be changing.

The greatest challenges to control of Chagas disease in Central America are non-eliminable, widespread vectors and underfunded and irregularly decentralized health systems. Although the disease has been targeted for elimination [18], a more realistic approach is to prepare for permanent control in the region [19]. The success of vector control efforts in reducing household infestation and disease prevalence have made vector bugs and patients less visible and made the interventions less likely to be prioritized for government budgets in the future. Prospects for external funding are not good, since international aid agencies are often attracted to health problems which are eliminable or reducible to a great extent in a short time. Thus, Chagas disease control strategies need to be extraordinarily cost-effective and sustainable, and intervention models should be simple enough to be readily integrated and monitored in local health systems at different stages of decentralization. Although in Guatemala, El Salvador, and Honduras community-based vector surveillance for Chagas disease is part of the local health systems and functions with existing human resources and minimum costs, reductions in budget could affect availability of transportation and insecticide, and consequently health service responsiveness.

In the control of non-eliminable vectors, such as *T*. *dimidiata*, the roles of continued spraying of infested houses and alternative interventions must be determined. In our study, 33.5% of responses to infested households was by insecticide spraying in Guatemala, versus 95.8% in El Salvador and 84.0% in Honduras. This partly reflects periodic scarcity of insecticides in the Guatemalan Ministry of Health, but also a deliberate shift towards house improvement. Multiple cycles of insecticide spraying are effective in reducing household infestation [20], but are costly and difficult to sustain in the long run. Moreover, continuous application of insecticide might promote emergence of resistance in vectors. On the other hand, risk factors such as cracked mud walls, dirt floors, thatched roofing, and improperly tiled roofing [21] can be mitigated using locally available materials [22, 23]. The cost-effective approach for improving house structures and living conditions innovated by Guatemalan researchers was adapted by the country’s Ministry of Health [22, 23]. Also, local operational technicians developed an effective community organization approach which promotes engagement by the population and local government, and efficient implementation and scale-up of the house improvement method [5]. Evaluation of these efforts should also be part of the future research agenda.

This research found that consistent monitoring at the departmental level of the Ministry of Health makes a significant difference in health service responsiveness in community-based vector surveillance of Chagas disease. Other potential factors, such as the number of infested households, numbers of health personnel and community workers, distance from departmental health offices to health centers, and degree of decentralization of response seemed to have limited impact on health service responsiveness. Challenges related to these factors were met largely because of managerial efforts of the local health services in implementing short-term and long-term strategies. Basic management practices such as monitoring and supervision combined with thoughtful strategies can improve health service responsiveness in resource-limited settings.

## Supporting Information

S1 Table Results of community-based vector surveillance in the 12 study areas in Guatemala, El Salvador, and Honduras from 2008 to 2012. (XLSX)

S2 Table Results of mixed-effects multi-linear regression on health services’ response rates clustered by response pattern with potential determinants in community-based vector surveillance in Guatemala, El Salvador, and Honduras (N = 56). (XLSX)
